# Modeling adsorption and photocatalytic treatment of recalcitrant contaminant on multi-walled carbon/TiO_2_ nanocomposite

**DOI:** 10.1007/s11356-023-28852-8

**Published:** 2023-08-01

**Authors:** Kwena Yvonne Pete, John Kabuba, Benton Otieno, Aoyi Ochieng

**Affiliations:** 1 Department of Chemical and Metallurgical Engineering, Vaal University of Technology, Vanderbijlpark, South Africa; 2 Botswana International University of Science and Technology, Private Bag 16, Palapye, Botswana

**Keywords:** Adsorption, Heterogeneous, Kinetics, Nanocomposite, Photocatalysis, Tetracycline

## Abstract

A nanocomposite photocatalyst consisting of titanium dioxide (TiO_2_) supported on multiwalled carbon nanotubes (MWCNTs) has been successfully prepared and used for the treatment of wastewater contaminated with tetracycline (TC), a recalcitrant antibiotic pollutant. The TiO_2_/MCNT composites were prepared by a simple evaporation-drying method. The properties of MWCNT/TiO_2_ were optimized by dispersing different amounts of TiO_2_ onto MWCNT. The structural and optical characteristics of the nano-engineered photocatalyst composite were characterized using scanning electron microscopy (SEM), X-ray diffraction (XRD), and Fourier-transform infrared spectroscopy (FTIR) techniques. Photocatalytic degradation of TC was conducted in a quartz glass reactor. Different kinetic models were used to demonstrate the governing mechanisms. The findings revealed that the TiO_2_/MWCNT composite had enhanced photocatalytic activity (95% TC removal) compared to TiO_2_ (86% removal). The photocatalyst nanocomposite exhibited overall pseudo-second-order reaction kinetics and favored the Langmuir adsorption isotherm. Although up to 95% degradation of TC was achieved, only 75% of it was mineralized as a result of the formation of stable refractory intermediates.

## Introduction

Water is a critical component of environmental, social, and governance (ESG) and sustainability challenges faced globally. Finding solutions for wastewater management that are environmentally sustainable remain the main challenge given the increasing demands of the ever-growing world population (Boffo and Patalano 2020; Amdeha and Salem 2022). The presence of inorganic and organic pollutants from human, industrial, and agricultural activities makes the water supply challenges complex (Luo et al. 2023). Every day new recalcitrant chemicals such as pharmaceuticals and their metabolites are identified in the water environment posing a serious risk to aquatic ecosystems (Liu et al. 2018). Antibiotics are among the significant pharmaceutical components and have been widely used in veterinary and human medicine for treating and preventing bacterial infections (Xu et al. 2017). It has been reported that the concentration of antibiotics in hospital and pharmaceutical wastewater can be as high as 500 mg/L and between 100 ng/L and 6 μg/L in untreated domestic wastewater (Cao et al. 2018; Wang et al. 2016a, 2016b). Tetracycline (TC) is one of the most extensively produced, used, and effective antibiotics all over the world. Due to its high chemical stability, tetracycline poses serious harm to humans and the environment. Emerging recalcitrant contaminants such as TC possess low biodegradability and are not amenable to conventional biological treatment methods (Kumar et al. 2019).

Several treatment methods, such as adsorption, ion exchange, ozonation, membrane separation, Fenton, electrochemical oxidation, and chemical precipitation, have been used to treat antibiotic-contaminated wastewater with varying degrees of success and drawbacks (Ji et al. 2023; Abou-Hadid et al. 2022). Heterogenous photocatalysis, which is an advanced oxidation process (AOP), has attracted considerable interest as a unique green purification technology due to the degradation of pollutants adsorbed on the surface of the photocatalyst that can be simultaneously regenerated under light irradiation. Nanosized photocatalyst composites are promising due to their high performance and unique properties (Luo et al. 2019a, 2019b). Nano-photocatalyst composites exhibit not only remarkable photocatalytic properties but also strong adsorption capacity due to their extraordinarily high surface area-to-mass ratio. Since photocatalysis is a surface reaction that occurs mostly on/near the surface of the catalyst, contaminants are removed more effectively if they can easily diffuse to the photocatalyst’s surface. Thus, the adsorption process may affect the performances of catalysts, particularly nano-photocatalysts (Luo et al. 2019a, 2019b; Zhou et al. 2019).

Heterogeneous photocatalysis using TiO_2_ and UV light is a cost-effective and eco-friendly treatment technology for the removal of recalcitrant compounds and to increase the biodegradability of persistent pollutants (Zhou et al. 2019). TiO_2_ is the leading photocatalyst due to its high biological and chemical stability, relatively low cost, good photoactivity, and low toxicity. Several studies have investigated the heterogeneous UV/TiO_2_ photocatalysis of many emerging contaminants (Luo et al. 2019a, 2019b; Yu et al. 2021; Xue et al. 2011). It should be noted that the mass transfer limitation has to be minimized for an effective TiO_2_-based heterogeneous photocatalysis application in wastewater treatment. The main critical issue is to recover the TiO_2_ particles from the treated wastewater. In addition, the efficiency of TiO_2_ has been unsatisfactory given the rapid electron-hole pair recombination (Otieno et al. 2017; Brooms et al. 2018). To enhance the photocatalytic activity of TiO_2_, it is crucial to reduce the recombination rate of photogenerated electron and hole pairs (Tolosana-Moranchel et al. 2017; Manassero et al. 2017). TiO_2_ can be supported onto a conductive material with a large specific surface area to overcome these challenges.

Multiwalled carbon nanotubes (MWCNTs) present the advantages of being easy to synthesize, high essential electrical conductivity, suitable specific surface area, and improved chemical and mechanical stabilities (Liu et al. 2019; Dutta et al. 2018). Thus, MWCNTs are very promising materials that can be composited with TiO_2._ Furthermore, it is assumed that supporting TiO_2_ onto MWCNTs can promote dispersion, induce charge transfer, and thus improve the photocatalytic activity of TiO_2_ for degrading recalcitrant emerging pollutants (Sun et al. 2018). Many researchers have combined adsorptive and photocatalytic means for the removal of aqueous contaminants by using engineered nanomaterials, including adsorbents and photocatalysts with environmental applications (Mohamed et al. 2022; Dubey et al. 2022; Luo et al. 2023). However, few studies have focused on how photocatalytic activity and adsorption work together. In addition, the design and use of effective TiO_2_/carbon material systems depend on a complete understanding of this interaction (Meng et al. 2022).

Previous studies on the integration of adsorption and photocatalysis have overlooked the role of adsorption kinetics when evaluating pollutant degradation by assuming the adsorption/desorption interaction is always at equilibrium (Luo et al. 2019a, 2019b; Xue et al. 2011; Martínez et al. 2011; Son et al. 2004; Ji et al. 2009). It is important to emphasize that the active species created by photo-induced carriers, which are crucial in the continuous degradation of the adsorbed reactant, hinder the adsorption/desorption interaction equilibrium from being attained in photocatalytic processes. According to Zhou et al. (2019), adsorption and photocatalytic degradation processes are indivisible and are widely used for pollutant degradation with practical environmental applications. To guide the development and improvement of nano-photocatalysts and advance the real-world applications of photocatalysis, a thorough understanding of the foundations of the modeling of the integrated adsorption and photocatalytic degradation of pollutants is required (Luo et al. 2019a, 2019b). The purpose of this research was to investigate the feasibility of using TiO_2_/MWCNT nanocomposite for the degradation of TC and to reveal the synergetic effect of surface adsorption and photocatalytic potential on the removal of a recalcitrant contaminant. Of special interest was the modeling of the photodegradation process and adsorption isotherms. Experiments were carried out in a systematic way to assess the removal of the TC contaminant by TiO_2_/MWCNTs under various conditions.

## Materials and methods

### Materials

TiO_2_ (P25, ca. 80% of anatase, and 20% of rutile) powder, supporting multiwalled carbon nanotubes (MWCNTs) with a specific surface area of 233 m^2^/g, an inner diameter of 3–5 nm and outer diameter of 5–8 nm, and recalcitrant emerging contaminant, tetracycline (TC, 95% purity), were purchased from Sigma-Aldrich, South Africa. All chemicals were of analytical grade and were used without further purification. Deionized water was used for the preparation of all aqueous solutions.

### Preparation and characterization of MWCNTs-TiO_2_ nanocomposite

According to Malikov et al. (2014), KMnO_4_ is an effective oxidizing agent since elements become more electronegative as the oxidation states of atoms increase. Furthermore, since oxidation occurs under mild conditions (i.e., lower temperatures), it is less costly than oxidation with HNO_3_ (Malikov et al. 2014). Pure MWCNTs were oxidized for 3 h in a 0.1 M KMnO_4_ solution with magnetic stirring at 80 °C. The solution was filtered, and the resulting residue was oven-dried at 100 °C for 12 h after cooling to room temperature and being rinsed with distilled water until pH 7 was reached. TiO_2_/MWCNT composites with varying TiO_2_/MWCNTs ratios were prepared by transferring weighed amounts of MWCNTs and TiO_2_ in ethanol and ultrasonically dispersing them for 2 h. The mixtures were then stirred magnetically for 30 min at 100 °C to enhance components’ interaction. The solutions were finally calcined at 450 °C for 2 h to produce the TiO_2_/MWCNT nanocomposites.

### Characterizations of the catalyst

Scanning electron microscopy (SEM), X-ray diffraction (XRD), and Fourier-transform infrared (FTIR) spectroscopy were used to examine the morphology, crystal phase, and nature of the surface molecular groups of the materials, respectively. X-ray diffraction (XRD, Rigaku – Ultima IV X-ray diffraction, scan speed 4.00=min, scan range 3–90) was used to investigate crystallinity and was performed using monochromatic Cu K radiation and a positional sensitive detector. FTIR spectra were recorded using KBr transparent discs at 64 scans between 4000 and 400 cm^−1^ with a resolution of 4 cm^−1^. The 105 N vacuumed-dried samples were pressed into a transparent disk with a diameter of 14 mm by applying force. The absorbance of KBr-sample discs was measured.

### Adsorption and photocatalytic experiments

**Fig. 1:**
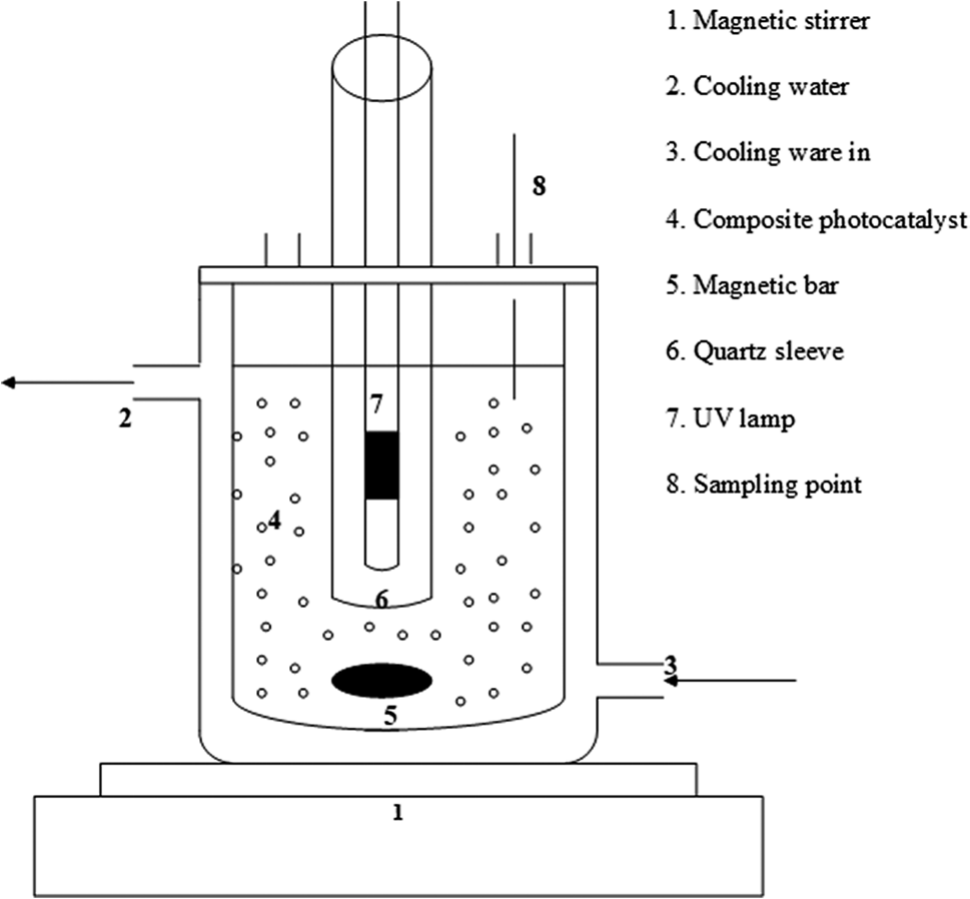
Schematic diagram of the photocatalytic reactor set-up

To investigate the adsorption and photocatalytic activity of the prepared TiO_2_/MWCNTs, tetracycline (TC) was used as a model recalcitrant contaminant. Experiments for photolysis, adsorption in the dark, and photocatalytic degradation by TiO_2_/MWCNTs were carried out in a 250-mL cylindrical batch reactor (Fig. 1) under continuous stirring. Adsorption tests were conducted in the dark, and the minimum time required to ensure equilibrium was set at 60 min. After reaching adsorption-desorption equilibrium, photocatalytic degradation was carried out using a 12-W Xenon lamp and UV light (= 254 nm). Several parameters were investigated, including composite composition, substrate pH, and initial concentration. Samples were collected at various time intervals during the experiments and centrifuged at 3500 rpm for 2 min to separate the solution from the composite photocatalyst. A UV-Vis spectrophotometer was used to measure the concentration of TC at the maximum absorption wavelength of 357 nm (Wu et al. 2020).

The initial TC concentration was set in the range of 10–100 mg/L at a composite photocatalyst dose of 1 g/L for the adsorption isotherm study. The adsorption amount (*q*_e_) retained per gram of composite photocatalyst at reaction time *t* was calculated as:

1 $${q}_{\mathrm{e}}=\frac{\left({C}_{\mathrm{i}}-{C}_{\mathrm{e}}\right)}{m}\ \mathrm{V}$$

where *C*_i_ and *C*_e_ (mg/L) are the TC concentration at initial and equilibrium time; *V* is the total solution volume (L); and *m* is the mass of TiO_2_/MWCNT added in the solution (g).

## Results and discussion

### Characterization results

**Fig. 2:**
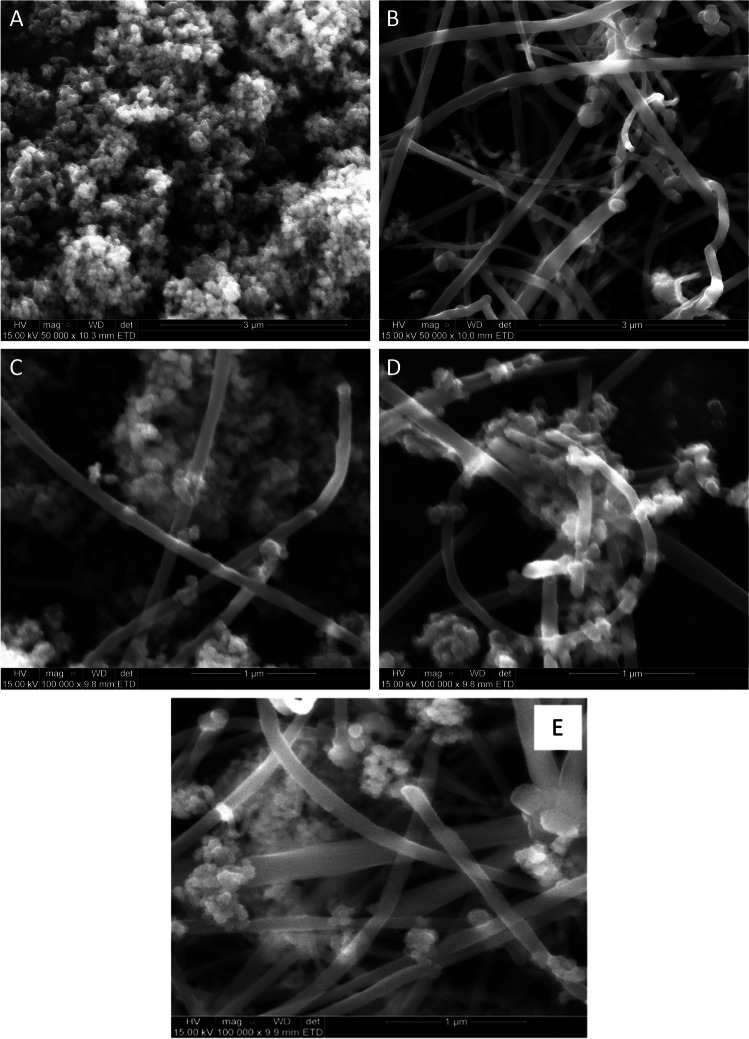
SEM images of **A** TiO_2_, **B** MWCNTs, **C** 0.05% TiO_2_/MWCNTs, **D** 1% TiO_2_/MWCNTs, and **E** 5% TiO_2_/MWCNT composite

Figure 2 shows SEM images of TiO_2_ (P25), MWCNTs, and MWCNT/TiO_2_ composite photocatalysts (at various ratios). TiO_2_ and MWCNTs were found to have spherical and tubular structures, respectively. SEM images of MWCNT/TiO_2_ revealed similar morphologies for all ratios, demonstrating TiO_2_ interaction with MWCNTs. MWCNTs are seen to be deposited evenly and tightly around the surface of TiO_2_. Aggregation was observed in Fig. 2 at higher MWCNT loadings, and this could be caused by the high surface energy of the MWCNTs. The structural analysis revealed that MWCNTs were coated on the surface of TiO_2_ nanoparticles, despite the presence of agglomerates of TiO_2_, which was likely due to the relatively high TiO_2_ content (5%) in the nanocomposite. A crystalline material is distinguished by its orderly and continuously repetitive arrangements of atom planes in its crystal lattice (Ali et al. 2021).

**Fig. 3:**
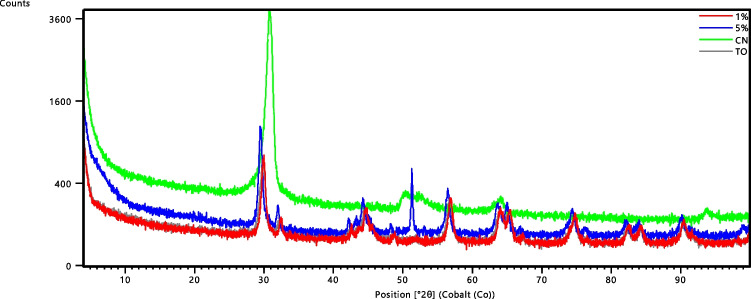
XRD patterns of TiO_2_, MWCNTs, and TiO_2_/MWCNT composites

The XRD pattern of the o-MWCNTs revealed a high intense peak at approximately 30.95° and a low intense peak at 48.5°, as shown in Fig. 3. The peak at 30.95° in the XRD pattern for o-MWCNTs corresponds to the plane (002) of graphite hexagonal structure, and the next peak at around 48.5° corresponds to the plane (1.00) of graphite hexagonal structure (Natarajan et al. 2017; Caudillo-Flores et al. 2016). The XRD pattern for TiO_2_ nanoparticles showed several anatase phase peaks at 29.9°, 43.6.78°, 56.8°, 65.3°, and 74.7° (Meng et al. 2022). The presence of the rutile in TiO_2_ was also indicated by the peaks appearing at 42.6°, 52.04°, 64.42°, 78.21°, 89.51°, and 96.66° (Zhang et al. 2019). The MWCNT peak is not visible in the XRD pattern of TiO_2_/MWCNTs due to TiO_2_ being more abundant than MWCNTs. Furthermore, the crystalline extent of MWCNTs is much lower than that of TiO_2_, resulting in the shielding of MWCNTs peaks by TiO_2_ peaks (Shahnazi et al. 2020). These findings confirmed the successful synthesis of the TiO_2_/MWCNT nanocomposite.

**Fig. 4:**
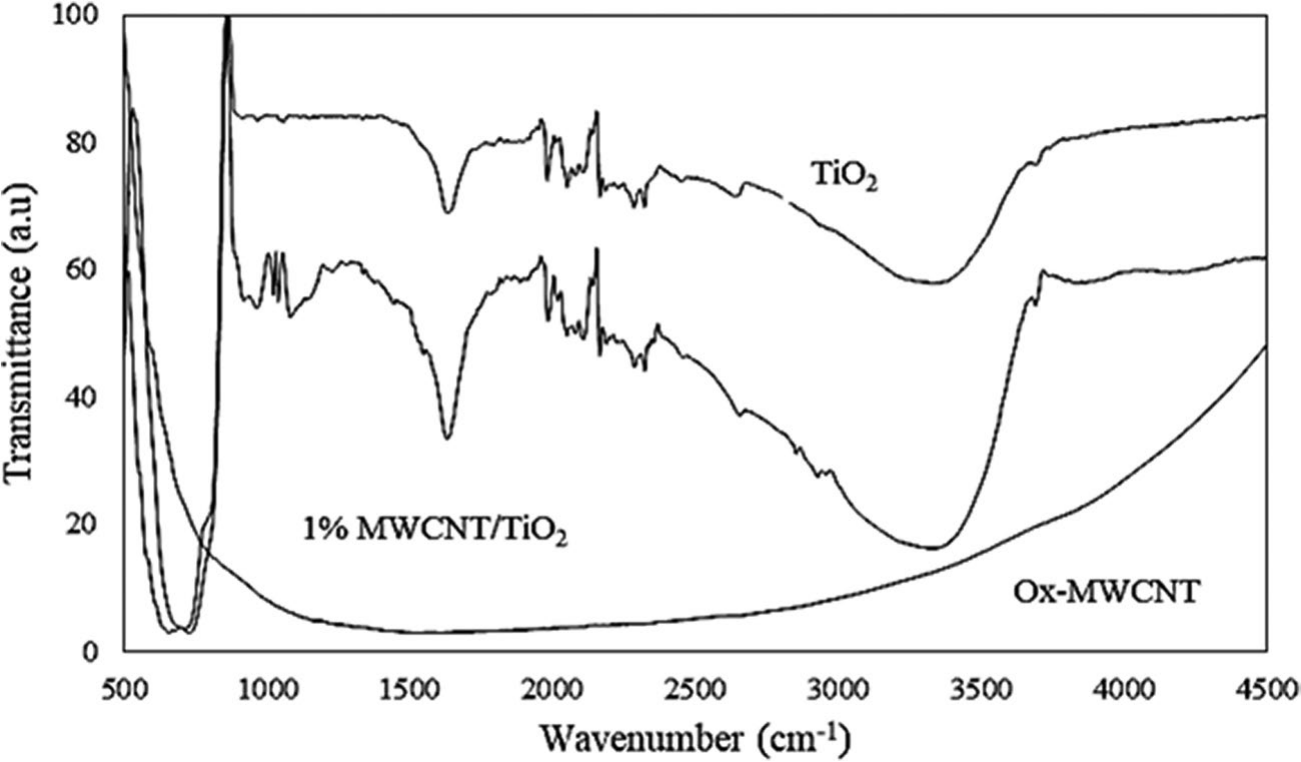
FTIR spectra of MWCNTs, TiO_2_, and TiO_2_/MWCNT composite

Figure 4 depicts the FTIR spectra of TiO_2_, MWCNTs, and TiO_2_/MWCNT composite particles. TiO_2_/MWCNT composite and TiO_2_ had nearly identical spectra. Fourier-transform infrared spectroscopy (FTIR) confirms the relationships and functions of various materials (Meng et al. 2022). The stretching and bending vibrations of the surface-OH group cause the adsorption peaks at 3480 cm^−1^ and 1600 cm^−1^ (Shahnazi et al. 2020). The peak due to Ti-O and O-TiO stretching and binding modes appears around 500 cm^−1^. In the MWCNT/TiO_2_ composite spectrum, the peak at 500 cm^−1^ is sharper than that of pure TiO_2_, which could be attributed to differences in crystallinity and size (Esfe et al. 2022).

### Adsorption and photocatalytic degradation studies

**Fig. 5:**
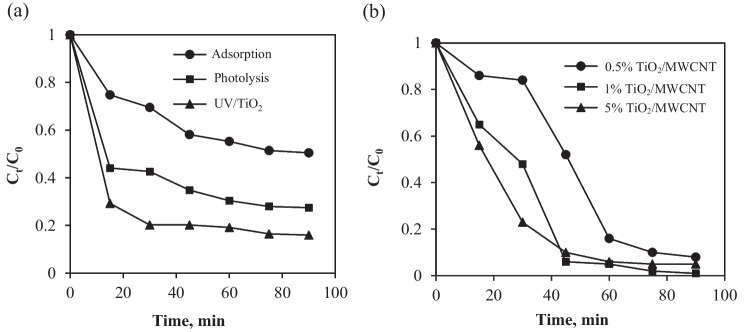
**a** Control experiments for the treatment of TC under adsorption, photolysis, and photocatalysis, and **b** photocatalytic degradation of TC in the presence of different ratios of the TiO_2_/MWCNT photocatalyst under irradiation. Conditions: catalyst dose 1 g/L, TC concentration 20 mg/L, and pH 6.5

Approximately 85% of TC was removed during the 90-min reaction time through adsorption and photodegradation, while 73% and 54% were removed in the photolysis and adsorption systems, respectively (Fig. 5a). The major challenges of TiO_2_ are aggregation and agglomeration, which affect light absorption and photoactivity. Furthermore, TiO_2_ photoactivity at low wavelengths is limited by the rapid recombination of photogenerated electron-hole pairs, as well as its large band-gap value of 3.2 eV. Carbon nanotubes used as support materials addressed these issues, preventing electron/hole pair recombination (Fig. 5b). This allowed more charge carriers to successfully diffuse into the surface, increasing TiO_2_ absorption affinity towards the target contaminants while also preventing TiO_2_ particle aggregation and agglomeration (Wang et al. 2015).

MWCNTs can also absorb photons, become excited, and inject electrons into the TiO_2_ conduction band, causing highly reactive hydroxyl and superoxide radicals to form (Czech and Tyszczuk-Rotko 2018). A decrease in photoactivity was observed at higher MWCNT percentages due to the TiO_2_ surface being completely covered with MWCNTs. Figure 5b shows the results of the TC removal obtained by varying the amounts of MWCNTs added to the TiO_2_ catalyst. The prepared 1% TiO_2_/MWCNTs composite photocatalyst achieved 95% efficiency in TC degradation. The removal of almost half of TC occurred within the first 15 min of reaction time for the 5% TiO_2_/MWCNTs composite photocatalyst, and this is attributable to more adsorption taking place than photodegradation.

### Effect of catalyst loading on adsorption and photocatalytic degradation

**Fig. 6:**
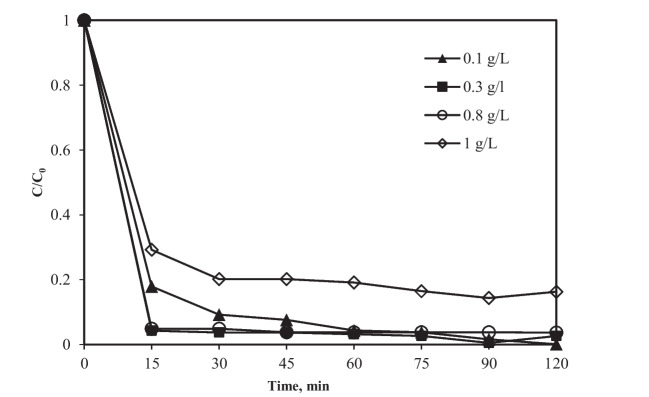
Effect of TiO_2_/MWCNT nanocomposite loading on TC removal. Conditions: TC concentration 20 mg/L, and pH 6.5

The effect of optimal loading of TiO_2_/MWCNT nanocomposite on photocatalytic degradation of the recalcitrant compound was investigated at TC concentration of 20 mg/L, pH value of 6.5, and irradiation time of 120 min (Fig. 6). Figure 6 shows that TC photocatalytic degradation efficiency increased as photocatalyst loading increased, with the highest removal observed at 0.1 g/L after 90 min. The increase in the number of adsorption and photocatalytic sites available on the surface of the TiO_2_/MWCNT nanocomposite can be attributed to this result. In a similar reaction time, there was no further improvement for loadings greater than 0.1 g/L. High photocatalyst loading can cause light scattering and consequently reduce the specific activity of the photocatalyst. Furthermore, particle agglomeration at high loading can interfere with the homogeneous-like structure of the suspension, thereby reducing the number of active sites (Wang et al. 2009).

### Effect of solution pH on adsorption and photocatalytic degradation

**Fig. 7:**
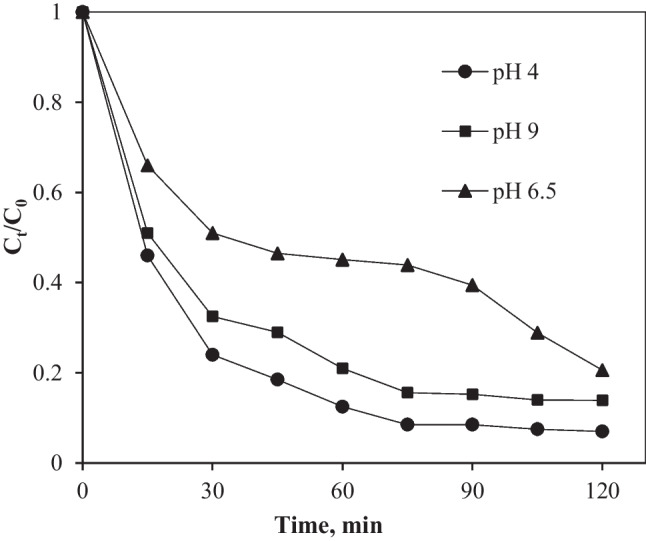
The effect of initial pH on TC removal. Conditions: catalyst dose 1 g/L, TC concentration 20 mg/L

The TC degradation was least effective at alkaline pH and more effective at neutral and acidic pH conditions (Fig. 7). At the alkaline pH of 9, the adsorption and photocatalytic degradation of TC were hampered, leading to an overall removal of 51% (Fig. 7). At the acidic pH of 4, the adsorption and degradation efficiencies were greatest (93% TC removal). The initial pH of the solution is critical in the degradation and adsorption of the target organic compounds. The pH of the surface of the photocatalyst affects its electrical charge properties and determines the ionization state (Wang et al. 2009; Yuan et al. 2022). The observed adsorption and photocatalytic behavior can be attributed to a combination of TC species speciation, TiO_2_/MWCNT surface charge properties, and possible complexation between TC and TiO_2_/MWCNTs (Ahmadi et al. 2017). The increase in adsorption and photocatalytic degradation at acidic and neutral pH can be attributed to the point-of-zero charge (PZC) of the TiO_2_/MWCNT nanocomposite and TiO_2_, which have been reported to be approximately 6.6 (Saleh and Gupta 2012) and 6.5, respectively (Wang et al. 2009).

The decrease in adsorption and degradation efficiency at pH 9 can be attributed to either TC or TiO_2_ changing to an anionic form, in which case repulsive electrostatic forces prevented TC sorption onto TiO_2_ (Ahmadi et al. 2017). Furthermore, changes in solution pH affect the formation of hydroxyl radicals on TiO_2_, and the type of products and reactants via the corresponding pKa values (Yuan et al. 2016). TiO_2_ nanoparticles have a positively charged surface in acidic pH conditions and a negatively charged surface in the alkaline pH range (pH ZPC = 6.5). Tetracycline exists as neutral (H_2_TC) in pH values between 3.3 and 7.68, cationic (H_3_TC^+^) in pH values less than 3.3, and anionic (HTC^−^ and TC^−2^) in pH values greater than 7.68 (Liu et al. 2015). As a result of the surface charges of TC and TiO_2_, at a pH range of 3.3 to 7.68 TC appears in molecular form favoring its sorption, thus improving the photocatalytic process.

### Adsorption and photodegradation kinetics

To obtain the heterogeneous photodegradation rate constant, the first-order kinetic (Eq. 2), second-order (Eq. 3), and modified Elovich (Eq. 4) models were used to fit the experimental data:

2 $$\frac{\mathrm{d}{C}_{\mathrm{t}}}{\mathrm{d}\mathrm{t}}=-{k}_1{C}_{\mathrm{t}},$$

3 $$\frac{\mathrm{d}\left({C}_{\mathrm{t}}\right)}{\mathrm{d}\mathrm{t}}=-{k}_2{C_{\mathrm{t}}}^2,$$

4 $$\frac{\mathrm{d}\left({\mathrm{C}}_{\mathrm{t}}\right)}{\mathrm{d}\mathrm{t}}=-{k}_{\mathrm{e}}{C}_0\exp \left(\upbeta \left(\left({C}_{\mathrm{t}}-{C}_0\right)\right)\right.$$

where *C*_t_ and *C*_0_ are the TC concentrations (mg/L) at any time *t* and at time 0, respectively; *k*_1_, *k*_2_, (min^−1^), and *k*_e_ (min^−1^) are the degradation rate constants for the first-order, second-order, and modified Elovich models, respectively. *β* is a constant inversely proportional to the removal capacity (Massai et al. 2020; Mouhamadou et al. 2023).

When the three models were compared (Table 1), the second-order model simulated the experimental kinetic data the best, with *R*^2^ values of 0.979 and 0.968 at pH 4 and pH 6.5, respectively. The findings confirm that adsorption plays an important role in photodegradation and thus speeds up TC degradation by TiO_2_/MWCNT. The experimental data are well fit by the Elovich model (Table 1), suggesting that it might be used to explain the TC removal kinetics by the composite TiO_2_/MWCNT photocatalyst. When composite photocatalysts like TiO_2_/MWCNTs are utilized to degrade pollutants under light irradiation, the adsorption and photodegradation processes are crucial to the kinetics (Qing et al. 2022). The results support and clarify the underlying mechanism of the synergy between adsorption and degradation (Wei et al. 2021).

**Table 1:** The best-fit parameters of photocatalytic kinetics for TC removal at different pH conditions

Condition	Pseudo-first-order		Second-first-order		Elovich-second-order		
	*K*	*R*^2^	*K*	*R*^2^	Β	*K*	*R*^2^
pH 4	0.1202	0.9719	0.006	0.979	0.125	1.35E-9	0.951
pH 6.5	0.0558	0.958	0.0028	0.968	0.14	0.51	0.935

### Modeling adsorption isotherms

To investigate the adsorption equilibrium of TC, the Langmuir and Freundlich isotherms were used (Kinniburgh 1986; Luo et al. 2023). The Langmuir model predicts a homogeneous surface by monolayer adsorption in which all binding sites have the same adsorption affinity and no interactions between adsorbate and adsorbent are considered (Repo et al. 2010; Luo et al. 2023). The mathematical expression for the Langmuir adsorption isotherm is:

5 $${q}_{\mathrm{e}}=\frac{q_{\mathrm{m}}{K}_{\mathrm{l}}{C}_{\mathrm{e}}}{1+{K}_{\mathrm{L}}{C}_{\mathrm{e}}}$$

where *C*_e_ (mg/L) and *q*_e_ (mg/g) represent the equilibrium concentration of TC and the adsorption capacity of TiO_2_/MWCNTs, respectively. *q*_m_ (mg/g) is the maximum adsorption capacity of TiO_2_/MWCNTs, while *K*_L_ (L/mg) is the energy of the adsorption.

**Fig. 8:**
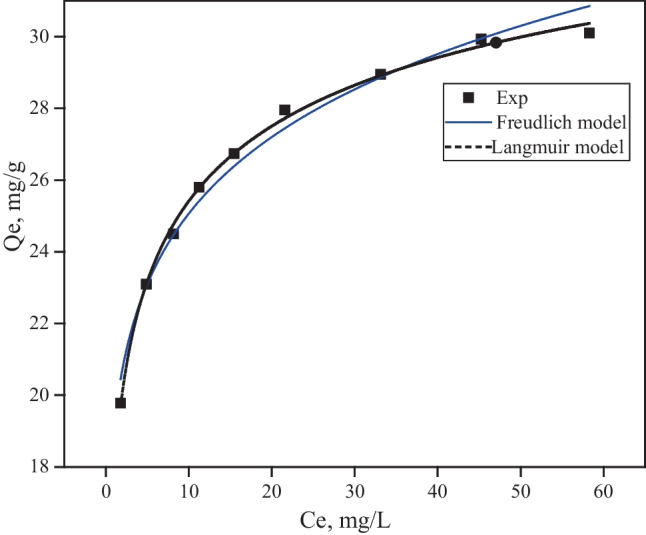
Modeling of TC adsorption isotherms

The Freundlich model assumes adsorption on a heterogeneous surface without adsorbent binding site saturation (Luo et al. 2019a, 2019b). The Freundlich model is defined as:

6 $${q}_{\mathrm{e}}={K}_{\mathrm{f}}{C_{\mathrm{e}}}^{1/{n}_{\mathrm{f}}}$$

where *K*_f_ (mg/g) is a unit capacity coefficient and *n*_f_ is the Freundlich parameter associated with the degree of system heterogeneity. The larger the *n*_f_ parameter (usually greater than unity), the more heterogeneous the system is (Luo et al. 2023). The Langmuir model correlated well with the experimental data, as shown in Fig. 8 and Table 2. The Langmuir model provided a more accurate estimate of the maximum adsorption capacity for TC without compromising the quality of the fit for the prepared composite photocatalyst. The Freundlich model demonstrated that the effect of surface heterogeneity results in a stronger adsorbate-adsorbent interaction.

**Table 2:** Isotherm parameters for the adsorption of TC onto the surface of TiO_2_/MWCNT

	Langmuir			Freundlich			
*R*^2^	*Chi*^2^	*K*_L (L/mg)_	*q*_e_ (mg/g)	*R*^2^	*K*_F_ (mg/g)	*1/n*	*Chi*^2^
0.997	0.04	0.806	39.7	0.983	19.11	0.11	0.22

### Mineralization and theoretical model mechanism of adsorption and photocatalytic degradation of TC

**Fig. 9:**
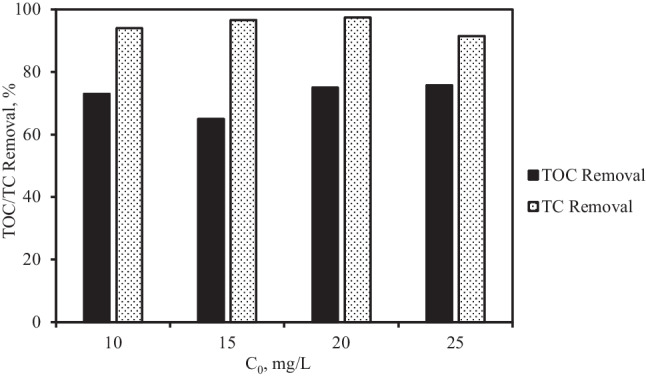
Comparison of TC removal and mineralization during photocatalytic degradation at different initial TC concentrations

The mineralization of TC was investigated by analyzing the results of total organic carbon (TOC) and TC removal at optimal conditions as:

7 $$Mineralization={TOC}_0-\left(\frac{TOC_t}{TOC_0}\right)\times 100$$

where TOC_0_ and TOC_t_ are initial and final TOC concentrations (mg/L), respectively. It was observed that the adsorption and photocatalytic process nearly removed all the TC, but with only 75% TOC removal (Fig. 9). The differences in TC and TOC removal can be attributed to TC compounds breaking down into stable intermediates (Nezamzadeh-Ejhieh and Shirzadi 2014; Zhou et al. 2020). The intermediates are refractory to further attack by the radicals resulting in incomplete mineralization and leading to the incomplete reduction in TOC (Wang et al. 2018).

**Fig. 10:**
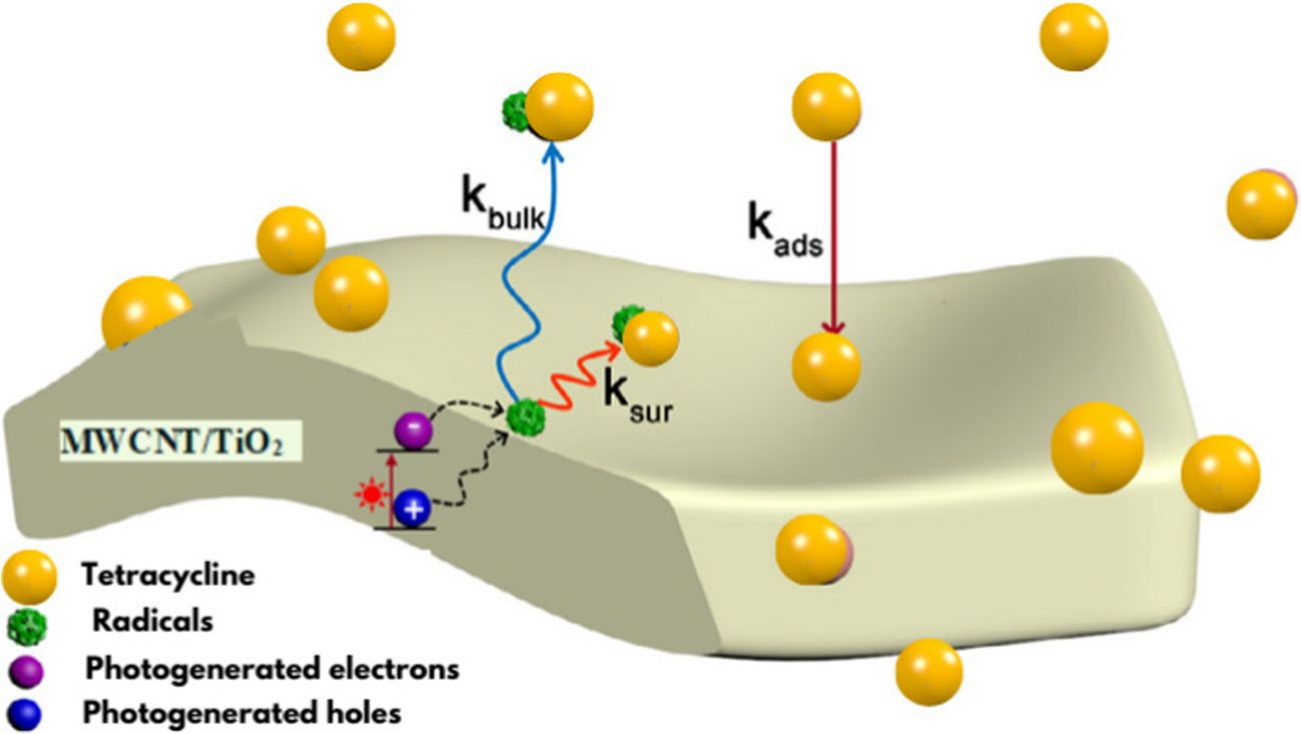
Theoretical representation of the adsorption and photocatalytic degradation of TC by MWCNT/TiO_2_ under UV light irradiation (Luo et al. 2019a, 2019b)

The rate-limiting processes are defined with a theoretical model in Fig. 10 to demonstrate the fundamental stages of TC removal by TiO_2_/MWCNTs and the relationship between the adsorption and heterogeneous degradation processes. Semiconductor photocatalysts such as TiO_2_ can absorb photons with ultra-bandgap energy, resulting in electron-hole pairs. According to the findings, the mechanism for the synergistic adsorption-photocatalytic degradation of TC by 1%TiO_2_/MWCNTs can be proposed. After UV irradiation, the TiO_2_/MWCNT surface (with a rate constant, *k*_sur_) rapidly adsorbs TC molecules (*k*_ads_), resulting in a rapid reaction between the tetracycline (with a rate constant, *k*_bulk_) and h^+^ dispersed on the composite photocatalyst surface. The separation of e^−^ and h^+^ is prompted by the consumption of h^+^. Meanwhile, the electron-hole pairs (e^−^ and h^+^) react with H_2_O and O_2_ leading to the formation of ^●^O_2_^−^ and ^●^OH radicals. The radicals then combine with TC molecules via redox reactions achieving effective degradation.

### Material stability and reusability

**Fig. 11:**
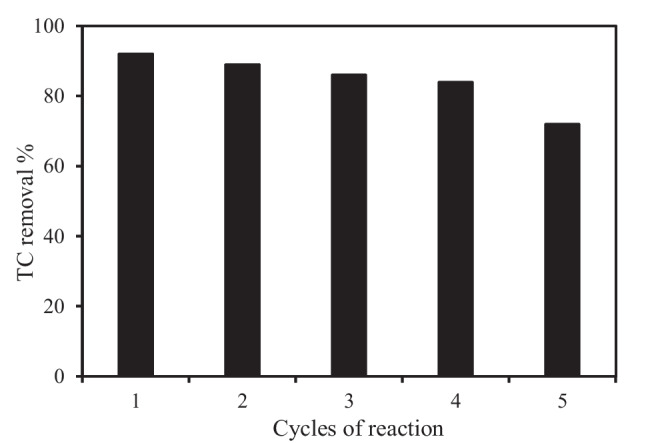
Reusability of TiO_2_/MWCNTs with consecutive runs. Reaction conditions: [TC] = 20 mg/L, TiO_2_/MWCNTs = 0.1 g/ L, time 120 min

Due to the high cost of catalyst synthesis, the reusability of the photocatalyst is critical from a financial perspective. The photocatalytic degradation performance of the MWCNT/TiO_2_ during 5 cycles was tested to assess the stability and reusability of the composite materials. At the end of each cycle, the photocatalyst was collected and used in the next cycle experiment. As illustrated in Fig. 11, the degradation rate could still exceed 90% during the first two cycles, and then, it slightly decreased in the ensuing cycles. The high TC removal of 72% after the fifth cycle indicated that the catalyst was stable and with high reusability.

## Conclusion

The MWCNT/TiO_2_ nano-engineered composite was successfully synthesized and demonstrated higher photocatalytic activity for the degradation of recalcitrant TC than pure TiO_2_. The resulting 1% TiO_2_/MWCNT composite photocatalyst achieved 98% removal efficiency in 90 min, with high reusability, exceeding 90% in the first two cycles and 72% after the fifth cycle. The composite performed best in weak acid and neutral solutions, according to pH studies. The second-order model best described the kinetics of the integrated adsorption-heterogeneous degradation of TC by TiO_2_/MWCNTs; however, the modified Elovich model also fairly described the process, confirming the importance of both adsorption and photodegradation. The Langmuir isotherm, with a maximum adsorption capacity of 39.7 mg/g, provided the best fit for the experimental data. The effect of surface heterogeneity was also seen, indicating that TC adsorption was intense and multilayered. The UV/TiO_2_/MWCNTs effectively removed TC, but TOC removal efficiency was only 75%. Differences in TC and TOC removal are due to TC molecules decomposing into stable intermediates. A theoretical model illustrating the basic steps of TC removal by TiO_2_/MWCNTs and the connection between the adsorption and heterogeneous degradation processes was used to describe the rate-limiting mechanisms. This study emphasized the strong synergistic effect of the adsorption properties of MWCNTs and the photocatalytic property of TiO_2_ (in the composite material) under UV irradiation. Adsorption was a critical process in controlling the kinetics of recalcitrant contaminant degradation, and as the surface adsorption of TC onto the substrate increased, so did the efficiency of TC degradation. As a result, the photodegradation process improved TC removal by MWCNT/TiO_2_ regenerating the adsorption sites. Due to the synergy between adsorption and photodegradation, the study shows that nano-engineered photocatalysts are a promising material for effective contaminant removal from aqueous solutions.

## Data Availability

All the datasets used in developing the manuscript are available on request.
